# Datamama, bringing pregnancy research into the future: design, development, and evaluation of a citizen science pregnancy mobile application

**DOI:** 10.3389/fdsfr.2023.1187023

**Published:** 2023-05-04

**Authors:** Eva Gerbier, Yvan Vial, Jardena Puder, Olivier Le Dizès, Magali Andrey, Amar Arhab, Antje Horsch, Valérie Avignon, Déborah Fort, Camille Deforges, Céline J. Fischer Fumeaux, Isabelle Henriot, Diana Pinto Pereira Goncalves, Léo Pomar, Guillaume Favre, Françoise Damnon, Hélène Legardeur, Gaëlle Mayor, Michael Ceulemans, Nohan Budry, Didier Page, Juergen Eisenberger, Olivier Liechti, David Baud, Alice Panchaud

**Affiliations:** ^1^ Service of Pharmacy, Lausanne University Hospital and University of Lausanne, Lausanne, Switzerland; ^2^ Materno-Fetal and Obstetrics Research Unit, Department “Woman-Mother-Child” Lausanne University Hospital, Lausanne, Switzerland; ^3^ Department Woman-Mother-Child, Obstetric Service, Lausanne University Hospital, Lausanne, Switzerland; ^4^ Department of Medecine, Endocrinology Diabetes and Metabolism Unit, Lausanne University Hospital, Lausanne, Switzerland; ^5^ Department Woman-Mother-Child, Neonatology Service, Lausanne University Hospital, Lausanne, Switzerland; ^6^ Institute of Higher Education and Research in Healthcare, University of Lausanne, Lausanne, Switzerland; ^7^ Clinic of Neonatology Department “Woman-Mother-Child”, Lausanne University Hospital, Lausanne, Switzerland; ^8^ School of Health Science (HESAV), University of Applied Sciences and Arts Western Switzerland, Lausanne, Switzerland; ^9^ Gaelle Mayor, Cabinet Médical Neuchâtel, Neuchâtel, Switzerland; ^10^ Teratology Information Service, Netherlands Pharmacovigilance Centre Lareb, Hertogenbosch, Netherlands; ^11^ Department of Pharmaceutical and Pharmacological Sciences, Clinical Pharmacology and Pharmacotherapy, KU Leuven, Leuven, Belgium; ^12^ L-C&Y, KU Leuven Child and Youth Institute, Leuven, Belgium; ^13^ School of Engineering and Management (HEIG-VD), University of Applied Sciences and Arts Western Switzerland, Yverdon-lesBains, Switzerland; ^14^ Chief Technical Officer at Avalia Systems, Yverdon-lesBains, Switzerland; ^15^ Institute of Primary Healthcare (BIHAM), University of Bern, Bern, Switzerland

**Keywords:** pregnancy, digital health (eHealth), pharmacoepidemiology, electronic databases, mobile application

## Abstract

**Background:** Pregnancy mobile applications (apps) have grown in popularity over the past decade, with some being used to promote study recruitment or health behaviors. However, no app serves as an all-in-one solution for collecting general data for research purposes and providing women with useful and desirable features.

**Aim:** To create and develop a Swiss pregnancy mobile app as an innovative means to collect research data and provide users with reliable information.

**Methods:** Determining the key features of the app involved a review of the literature and assessment of popular apps in the Swiss AppStore. A team of engineers developed the app, which includes a pregnancy timeline, questionnaires for data collection, medical and psychological articles and a checklist with appointment reminders. The content was written and reviewed by healthcare providers considered experts in the topics adressed. The questionnaires are distributed based on the user’s gestational age, by a chatbot. The project was authorized by the ethics commission in the canton of Vaud. An online survey of ten questions, advertised on Datamama’s home screen, was conducted to assess the users’ use of the app (27.11- 19.12.2022).

**Results:** A review of 84 articles and 25 popular apps showed the need for a comprehensive pregnancy app. The development of Datamama took 2 years and included the creation of 70 medical and psychological articles and 29 questionnaires covering 300 unique variables. Six months after the launch, there were 800 users with a 73% average participation rate in the questionnaires. Sixty-five women completed the survey, with 70.8% using the app once to multiple times per week. The primary reason for using the app was to help research by answering the questionnaires, followed by access to reliable medical information. The reason most frequently ranked first for using the app was to help research by answering the questionnaires (42/65, 67% of women rated it first), followed by access to reliable medical information (34/65, 54% women rated it second). Women rated the information as clear, understandable, and interesting with a trust rating in data handling at 98.5%. The average grade for recommending the app was 8/10, with suggestions for increasing the amount of medical content and tailoring it based on gestational age.

**Conclusion:** Datamama is the first pregnancy app to address the needs of both patients and researchers. Initial feedback from users was positive, highlighting future challenges for success. Future work will consist in improving the app, validating the data and use it to answer specific pregnancy-related research questions.

## 1 Introduction

Pregnant women’s health has been negatively impacted by their frequent exclusion from clinical trials and by the lack of sufficient funding for research in this area (Fisk and Atun, 2009; RAND Europe, 2020; Sample, 2022). Indeed, since the thalidomide scandal in the 1960s, fear and caution have prevailed regarding exposure to medication during pregnancy. While significant progress has been made towards reaching an equal proportion of men and women in clinical trials, pregnant women have maintained their *status quo*. In a review of all ongoing trials between 2013 and 2014 (Scaffidi et al., 2017), less than 0.5% were specifically designed for pregnant women. In addition, pregnancy and lactation were the most common exclusion criteria in a review of clinical trials on 38 new drugs approved by the Food and Drug Administration (FDA) between 2014 and 2017 (Duggal et al., 2021). Many researchers have called for the creation of frameworks to ensure the fair inclusion of pregnant women in clinical trials (Lyerly et al., 2008; White, 2015; van der Graaf et al., 2018; NHS Health Researsh Authority, 2022). In the meantime, post-authorisation observational data are still the leading source of information for research on medication safety and use during pregnancy. More recently, based on the observation that between 50% and 75% of women report using a pregnancy application (app) (Lee and Moon, 2016; Lupton and Pedersen, 2016; Özkan Şat and Yaman Sözbir, 2018), researchers have tried to use these devices as new means for study recruitment and data collection. As an example, in 2017, pregnant women being recruited through an integrated healthcare delivery system, Kaiser Permanente Washington (KPW), were asked to download an app and complete questionnaires on it (Rothschild et al., 2022). The authors compared the two data sources and highlighted the app’s ability to gather information on over-the-counter drugs, sensitive behaviours (e.g., alcohol and illicit drug use) and self-reported discontinuation of prescription medications. Pregnancy mobile applications have the potential to recruit many women rapidly, with geographically and socio-economic diverse backgrounds, though potentially younger and more often nulliparous (Wallwiener et al., 2016; Vignato et al., 2019; Rothschild et al., 2022). With regards to women’s expectations, they value personalised information, particularly on topics such as exercise, healthy eating, sleep (Halili et al., 2018), and on fetal development and bodily changes that occur during pregnancy (Sommer et al., 2017). Functionalities enabling them to schedule or remind them of their pregnancy appointments are much appreciated (Burgess et al., 2017; Halili et al., 2018). Overall, women report feeling supported by these apps as they can provide information and support when healthcare providers (HCPs) may not have time (Connor et al., 2018; Halili et al., 2018; Chan and Chen, 2019). They are also perceived as tools to connect with other women sharing the same experience (Connor et al., 2018; Biviji et al., 2021).

Surprisingly, while information is reportedly the most valued functionality, the lack of reliable sources, the sometimes inaccurate (Wallwiener et al., 2016; Bland et al., 2020; Brown et al., 2020; Womack et al., 2020; Brunelli et al., 2021; Muñoz-Mancisidor et al., 2021) or even impartial information to promote industries’ personal interests (Rollins et al., 2023), have been identified as major concerns with pregnancy apps.

Furthermore, only few applications have been used to communicate information dedicated to medication safety in pregnancy. To our knowledge, the only pregnancy apps providing such information are intended for healthcare providers only (Drugs in Pregnancy Lactation, 2022; MommyMeds, 2022; Reprotox, 2022).

Overall, there are currently no applications that combine general pregnancy-related information and specific information on medication safety, as well as provide pregnant women with other functionalities they find desirable, and collect research data. This article describes the creation of a Swiss pregnancy mobile application (“Datamama”) as a comprehensive solution for gathering research data and communicating general evidence-based pregnancy information and medication safety information in a tailored manner for both patients and healthcare providers. The users’ first feedback on the application has been explored and is also presented.

## 2 Materials and methods

This paper outlines the different steps in the creation and development of a pregnancy mobile application aimed at collecting data on pregnant women for research purposes and providing them with reliable health information.

### 2.1 Ideation

In April 2020, we searched the literature for pregnancy applications restricted to the five previous years on Pubmed with the following keywords included in the title: “Pregnant women;” “Pregnancy;” “Childbearing;” and “mobile app;” “app*;” “ehealth;” “electronic health.” The articles were analysed to determine useful functionalities for the users and for the researchers. For the users, these functionalities were divided between recurring functionalities and original ones, present only in certain apps. For the researchers, they corresponded to those that could increase the data collection and its quality. A prisma flow diagram of the literature search and app search process is provided in Supplementary Material S1.

This process was also carried out on the most popular apps in French retrieved from the Swiss AppStore using the following keywords: “Grossesse;”“Enceinte;”“Maman;”“Bébé” (i.e., “Pregnancy;” “Pregnant;” “Mother;” “Baby”). Our inclusion criteria for further investigation of the apps were the relevance of the app’s name and popularity. To evaluate popularity, we established a score corresponding to the number of reviews on the store multiplied by the average grade of the app. The score was calculated for the first 100 apps. The 25 apps with the highest score were selected. Apps addressing the preconception period or other medical fields were excluded.

### 2.2 Sketching the app

**FIGURE 1:**
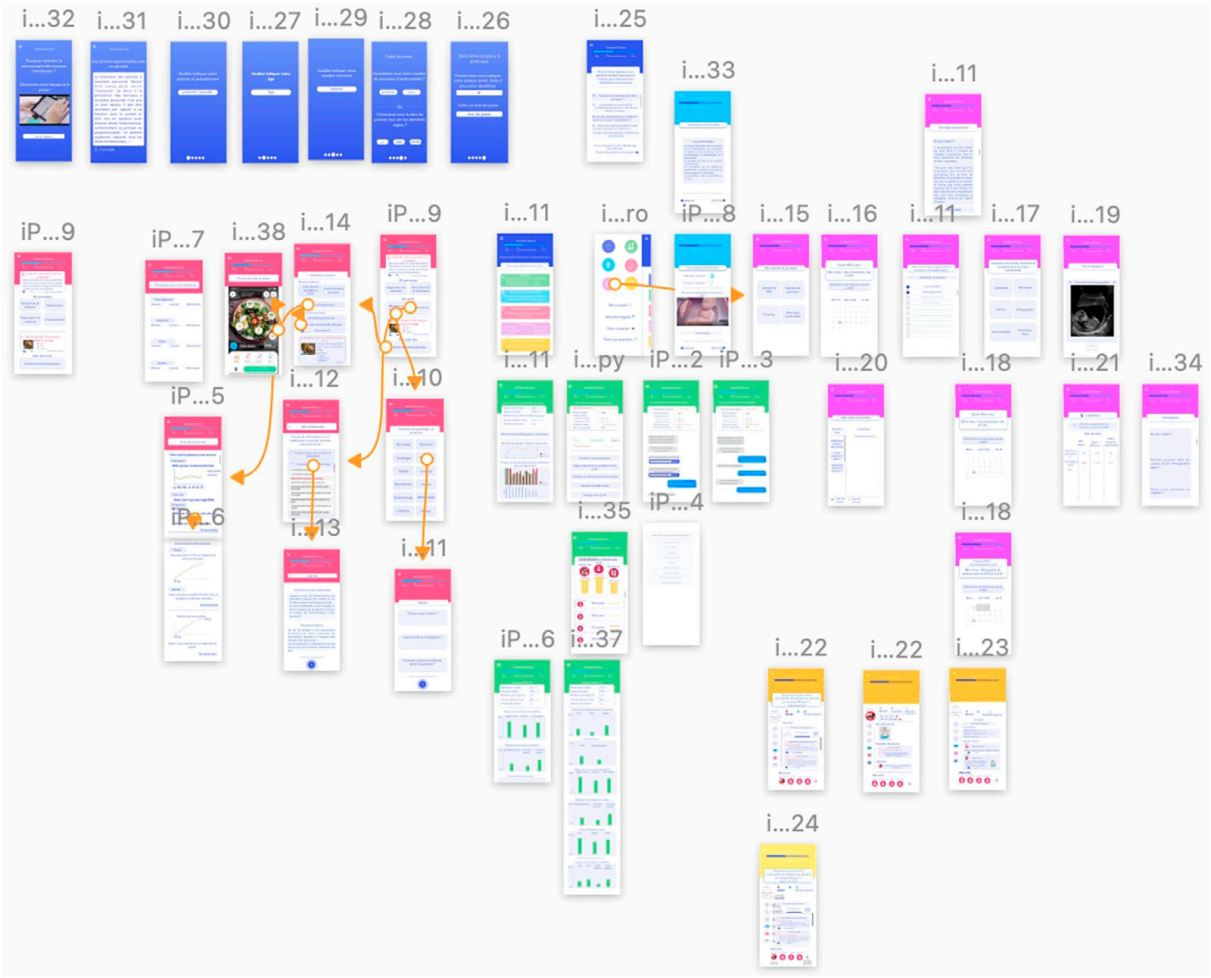
Wireframes of Datamama minimal viable product.

A first sketch of the app was conceptualised using the software “Sketch” (Sketch, 2022). The first step consisted in drawing the wireframes. A wireframe provides an overview of the architectural structure and organisation of an apps’ page without any styling or colouring detail yet (see Figure 1). Once the wireframes are drawn, the user flow can be determined. The user flow simply describes how one user can complete a specific task in the app, which should be as easy and intuitive as possible. They help determine how many pages will be necessary to move through the app.

### 2.3 Development

The application was developed by engineers from the Haute Ecole d’Ingénierie et de Gestion du canton de Vaud (HEIG-VD) between June 2020 and July 2022 (HEIG-VD, 2022). It was developed for use both for iOS and Android devices. Regular contact between the developers, who provided visual displays of the app and sometimes asked for clarifications, and the investigators, who gave feedback, occurred.

### 2.4 Content creation

The app content was designed with a pregnancy timeline in mind, covering appointments, exams, delivery, and early *postpartum*. Additionally, non-medical content was created to offer information on pregnancy-related topics such the most common perinatal mental health problem, travel, cosmetics, and animals. The content is displayed in the form of short medical/psychological articles (maximum 2-3 min reading time). Words that are not easily understandable are highlighted in the text and the user can find their definition by clicking on them. A search engine was developed to allow a keyword search to find a specific article. The first author (E.G.,) wrote some of the content which was reviewed by an obstetrician at the Centre Hospitalier Universitaire Vaudois (CHUV). Other experts were recruited to write and review content related to mental health, nutrition, physical activity, gestational diabetes, infectious diseases, and breastfeeding.

### 2.5 Questionnaires and data collection

A data collection tool was created to enable the distribution of questionnaires directly via the app. The questionnaires are dispensed by a chatbot taking the form of a casual conversation. They are sent at pre-determined time points based on the users’ gestational age, except for the subscription questionnaire, which is sent to all users when they start using the app for the first time. Users receive approximately one questionnaire per week. The questionnaires cover the following themes: 1) medical and obstetrical history, 2) obstetrical follow-up and outcomes, 3) medication and substance intake, 4) metabolic information (nutrition and physical activity), 5) sleeping habits, 6) environment (e.g., rural/urban living area, commute time, pets, etc.), and 7) breastfeeding.

To enhance the completeness of the data collection, weekly push notifications are sent to the user if she does not complete the form. The questionnaires contain only a limited number of items to minimize the necessary time to complete them. Moreover, to facilitate the response process, the most common response formats consist of tick boxes or in a predefined range of values when applicable, and very few of open-ended questions. Branching logics are used to reduce the number of items to complete. A gamification strategy is used to encourage participation. Gamification has been used previously in many mobile applications to promote health behaviours (Sardi et al., 2017). In our application, users receive points in exchange for the answers they provide based on their participation rate. These points allow to unlock a research status (for example: “Beginner,” “Expert” etc.). A contest is organized each month or every 2 months with a reward (i.e., massage, baby clothes, vouchers, cosmetics) for the best user(s). The best user is established on the participation rate and is reinitialized at the beginning of every contest. If multiple users have the same participation rate, a draw is organised between the different users. A leaderboard showing users with the highest participation rate is available and updated according to the timing of the contest. Finally, a visual display of the users’ and the community’s contribution are available (e.g., duration of participation; participation rate).

### 2.6 Ethical and legal issues

Datamama collects personal health related data and must therefore comply with the requirements of the Swiss Federal Human Research Act (HRA) (81 Health, 2011), more specifically to the Human Research Ordinance (HRO) (Fedlex, 2022). The use of a mobile application to collect non-anonymous sensitive data adds another layer of complexity compared to traditional recruitment methods. Indeed, users must provide their consent electronically instead of the traditional written consent. Therefore, women are requested to read the project information sheet directly on the app, agree electronically to each item of the consent and submit the form. Women subsequently receive via e-mail a pdf copy of both the information sheet and consent for personal storage. The contact details of one of the app’s investigators is provided in case of any question. All personal information, such as names, addresses, emails, are removed from the collected data to store de-identified data. The data are stored securely by the University of Lausanne’s (UNIL) servers. The Ethics Committee of the Vaud canton (CER-VD) granted authorization to complete this research project (Project-ID: 2021-00397, approved 12.05.2021). Finally, in addition to the ethical agreement, terms of use had to be drafted describing, amongst other things, guidelines for access and usage of the app, user privacy and data protection, and intellectual property rights.

### 2.7 Identity and design

Since aesthetics have been shown to play a major role in user perception and engagement (Halili et al., 2018; Thielsch et al., 2019; Brunelli et al., 2021), we asked a professional digital agency to help us redefine the apps’ identity and design.

### 2.8 Communication and promotion

Both digital and physical tools were used to promote the application and ensure successful user engagement. First, a website (https://datamama.ch) was developed by the same digital agency serving as an online showcase of the application (Datamama, 2022). A social network page on Instagram was created to share some short extracts of the app’s medical articles’ content and to promote the monthly contest. It was also helpful to locate influential personalities and pages with regards to pregnancy-related content and connect with them to gain visibility. A promotional, explanatory video featuring the principal investigators was developed to instill trust in the users. It was published both on the website and social media. In parallel, promotional flyers were displayed inside the CHUV’s maternity and sent to every pregnant woman who had an appointment at the obstetrics department since October 2022. Doctors and midwives at the CHUV were informed about the application during a medical seminar and asked to subsequently inform potential users.

### 2.9 Usability and feedback

A short online survey was conducted to assess the users’ use and perception of the application. The survey link was advertised on its home page. The survey was written in French, which is the current language of the application. It was available for 3 weeks, between 27 November 2022, to 19 December 2022 (the app was released in July 2022). Users had to provide an electronic consent when subscribing to the app. No further ethical approval was required to participate in the survey since the data collection was anonymous and not linked to the data collected on the app.

The survey consisted of ten questions (see Supplementary Materials S2, S3 for the survey questions in French and translated into English) addressing the app’s frequency of use (multiple choice question), the main reasons for using it (ranking question), the perceived quality of the information in the medical articles (rating questions), the frequency and clarity of the questionnaires (rating questions, 0 = “not frequent enough,” 5 = “ideal,” 10 = “too frequent”), and trust in data handling (yes/no question). Finally, women were asked whether they would recommend the app to a friend (rating question, 0 = “not at all” and 10 = “Strongly”) and what suggestions they had for the future developments of the app (open-ended question).

Results to multiple choice, ranking, rating, and yes/no questions are presented as absolute numbers, percentages, median scores and interquartile range (IQR). Results of the open-ended question are grouped into thematic key concepts.

## 3 Results

### 3.1 Ideation

After searching the literature for pregnancy applications, we retrieved 84 articles, and 18 articles were selected after title and abstract screening. In parallel, we downloaded and reviewed 25 apps with the highest popularity score on the Swiss AppStore. We classified the app’s functionalities for women (recurring ones and original ones) and for researchers (to increase data collection and its quality). Table 1 describes the classification of the app’s functionalities for both stakeholders based on the AppStore and literature review. Supplementary Material S4 provides the list of the 25 apps reviewed in the AppStore.

**TABLE 1:** Classification of the apps’ functionalities for users and researchers.

Functionalities for the users	
Recurring features	Original features
• Pregnancy timeline (number of pregnancy weeks/pregnancy trimesters; remaining time until delivery and due date)	• Calendar/checklist with next pregnancy appointments
• Medical information (bodily changes, fetal development, common pregnancy ailments)	• 3D fetal development visualisation
• Weight gain control (dietary recommendations, weight tracker)	• Cooking recipes for pregnancy
• Discussion forum (sorted by theme or by pregnancy age)	• Pregnancy diary
	• Goal setting for nutrition and physical activity

Functionalities for the researchers
• Possibility to have a voice/video recording of an answer
• Linkage with connected devices (i.e., smartphone, smartwatch)
• Provide standardized feedback based on the responses
• Interactive questions to verify and increase women’s knowledge
• Gamification strategy to increase responses

### 3.2 First sketch

After an iterative process between the author (EG) in charge of the project, the medical team (DB; AP) and the developers (DP; NB; OL; JE), a minimal viable product consisting of the app’s essential functionalities was agreed upon. These functionalities were selected among those retrieved from the literature and the app review (see Table 1). Thus, our first sketch comprised a pregnancy timeline indicating the user’s gestational age and a countdown until delivery, medical articles on various subjects of pregnancy and *postpartum*, a checklist with reminders of the next pregnancy appointments and exams, and questionnaires used for the data collection. Other functionalities such as 3D videos of fetal development, food recipes, and linkage with connected devices for health data collection were considered but not included due to time and cost restrictions. A pregnancy forum to share experiences with other pregnant women was also mentioned but removed from the initial sketch due to the monitoring efforts it would require. Figure 1 shows the wireframes of the minimal viable product. This sketch was presented to the developers in June 2020.

### 3.3 Development

The development of the minimal viable product took approximately 2 years, starting in June 2020 and ending in June 2022. It was prolonged due to the aesthetic and userflow modifications that had to be implemented following a professional review from a design agency (see part 7).

### 3.4 Content creation

**FIGURE 2:**
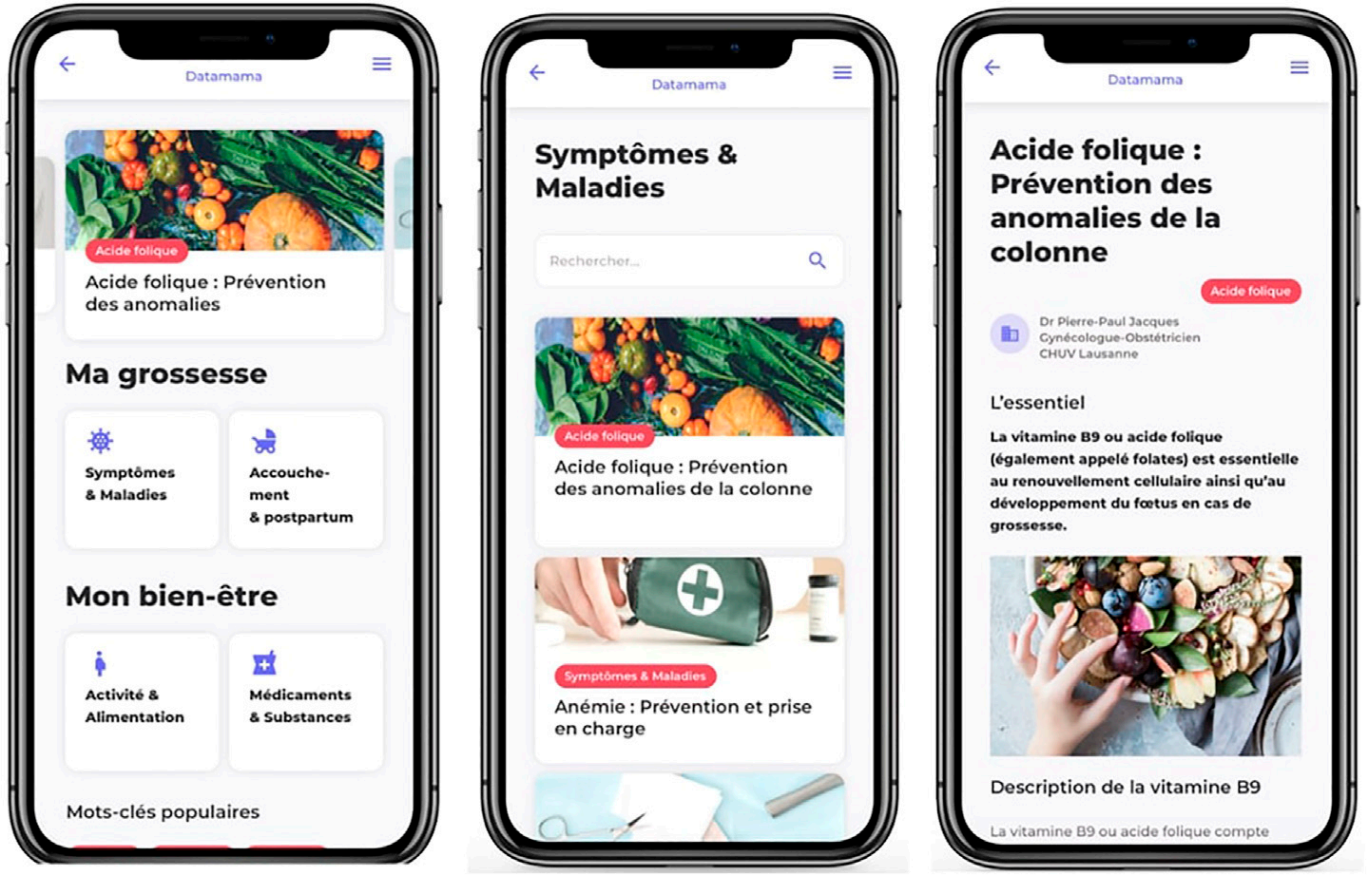
Screenshots of the information page and medical articles on Datamama. Left page is the information page, common to all users, divided in three main parts: at the top, a monthly blog; in the middle, “Ma grossesse” (“My pregnancy”) divided into two pages of medical articles: i.e., “Symptômes & Maladies” (“Symptoms and diseases”) and “Accouchement & *postpartum*” (“Delivery and Post-Partum”); and at the bottom, “Mon bien-être” (“My wellbeing”) divided into two pages of medical articles “Activité & Alimentation” (“Activity and nutrition”) and “Médicaments & Substances” (“Medication and substances”). The middle page consists of the “Symptoms and diseases”. Medical articles related to this category are sorted by alphabetical order. A search engine allows users to search by keywords. On the right page, an example of a specific article on the topic of folic acid is shown.

In total, more than 70 psychological and medical articles were written and will be regularly updated and completed by new articles. The content was subdivided into four categories: 1) Pregnancy follow-up, addressing common symptoms of pregnancy ailments and diseases of pregnancy (e.g., nausea and vomiting, constipation, pre-eclampsia); 2) Delivery and Post-Partum (until 8 weeks), focusing on the different stages of labour, anesthesia, complications of delivery, *postpartum*, breastfeeding, etc.,; 3) Medications and substances, providing advices on the treatment of common ailments of pregnancy (e.g., nausea and vomiting, pain, constipation) and describe the risks associated with smoking, or illicit drug use during pregnancy; and 4) Physical activity, Nutrition and Mental Health, addressing problems such as *postpartum* depression and perinatal support and providing recommendations on the latter topics. Figure 2 shows the information page with the different information categories, an example of a category and of a specific article on folic acid.

### 3.5 Questionnaires and data collection

**FIGURE 3:**
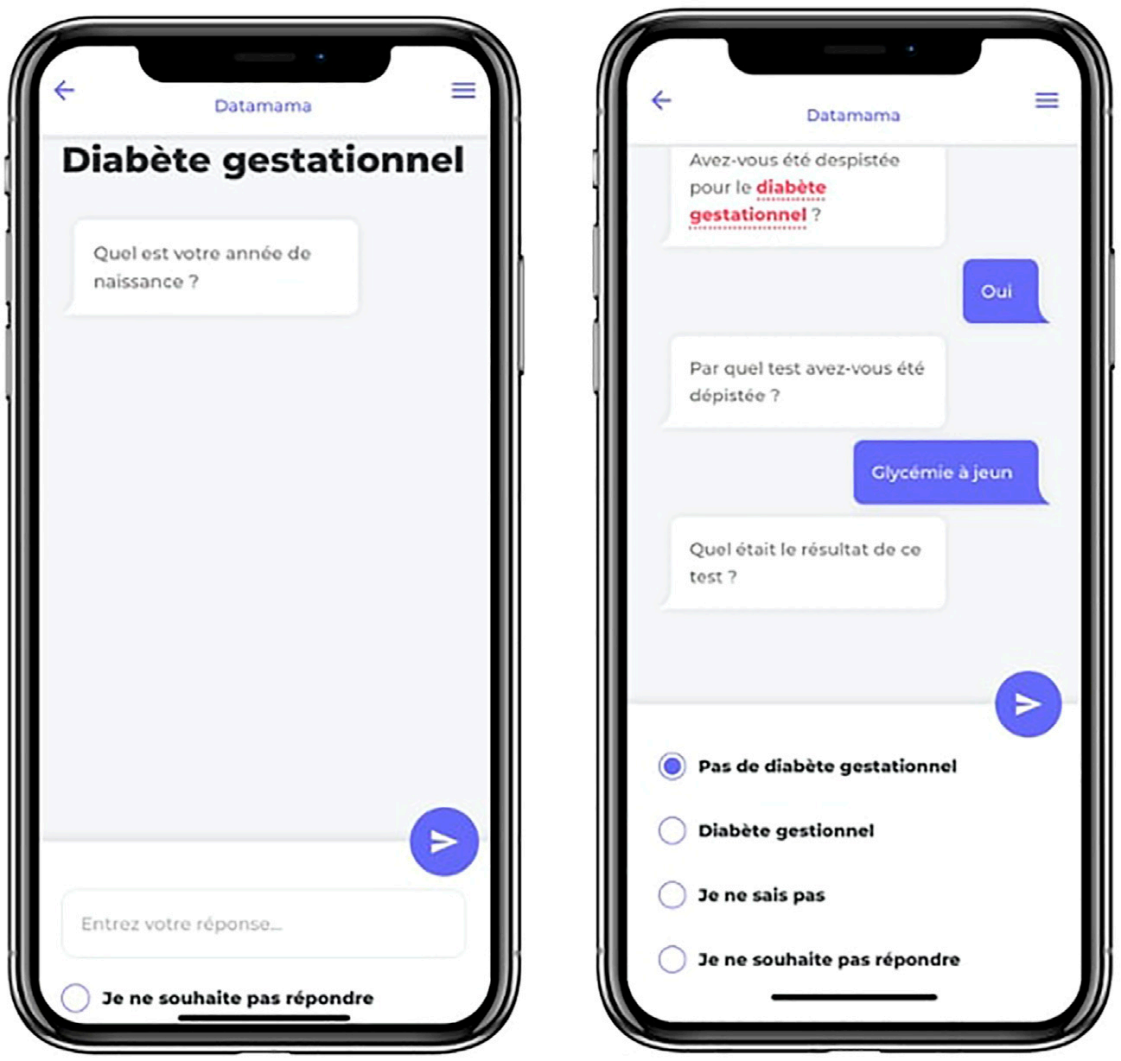
Screenshots of the survey tool on Datamama. The left page illustrates a question sent by the chatbot; the right page illustrates answers provided by the user. The different colours help distinguish between answers and questions, similarly to common messaging services.

**FIGURE 4:**
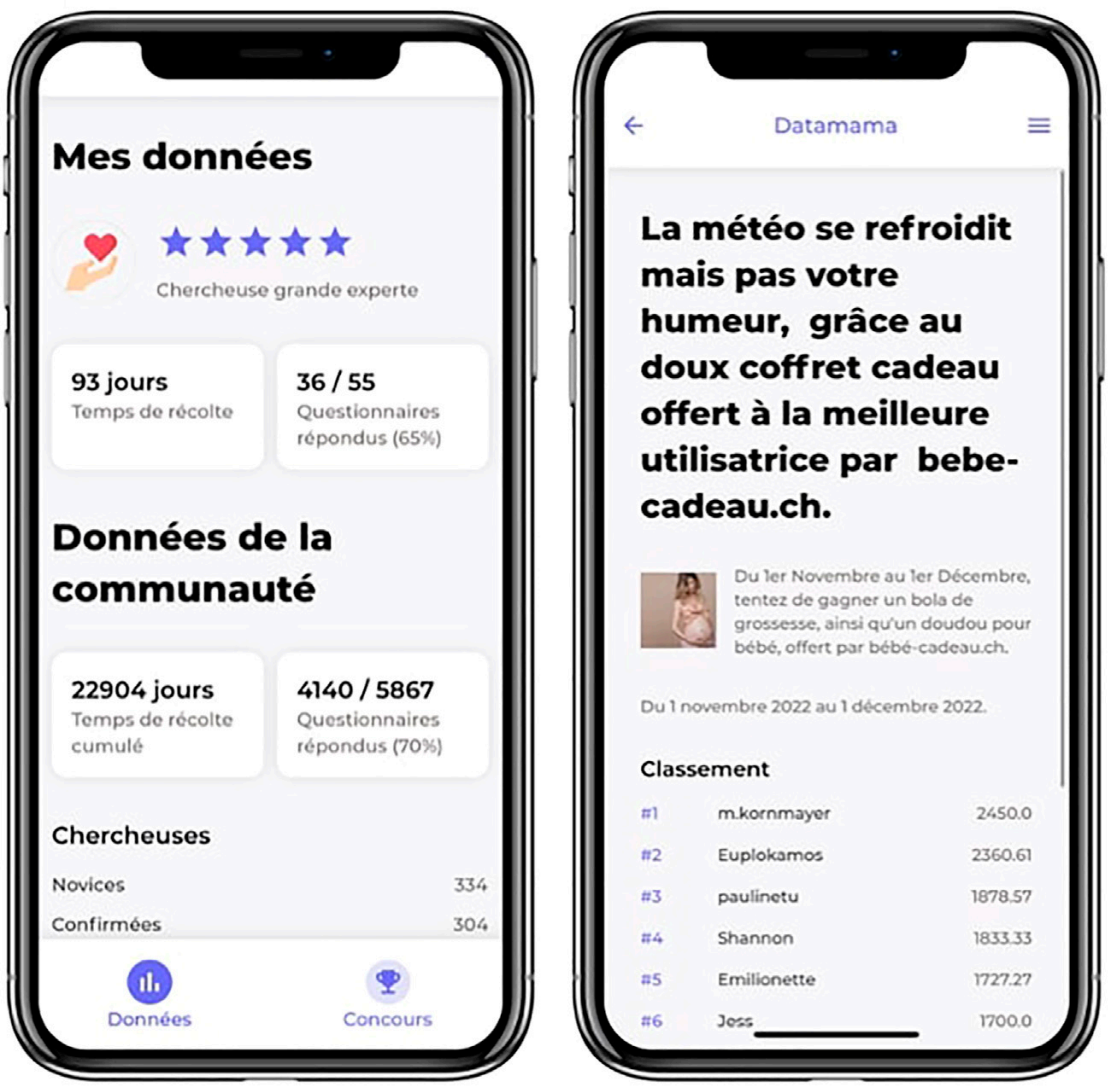
Screenshots of the gamification strategy and leaderboard. The left page illustrates the visualization of the user’s own data (i.e., research status, time since first collection, participation rate) and the community’s data (i.e., cumulated data collection time, participation rate). The right page illustrates an example of a monthly contest with the type of reward offered and overview of the users with the highest ranking. Users may choose to participate to the contest and to visualize this data or not.

In total, 29 questionnaires were developed covering 300 variables. Overall, in February 2023, 6 months after the app’s launch, 8,367 questionnaires had been sent and 6,163 answered (73% average participation rate). Due to technical constraints, the data retrieved from these questionnaires are not yet available for analysis. Figure 3 shows the questionnaire tool using a chatbot and Figure 4 the gamification strategy.

### 3.6 Identity and design

**FIGURE 5:**
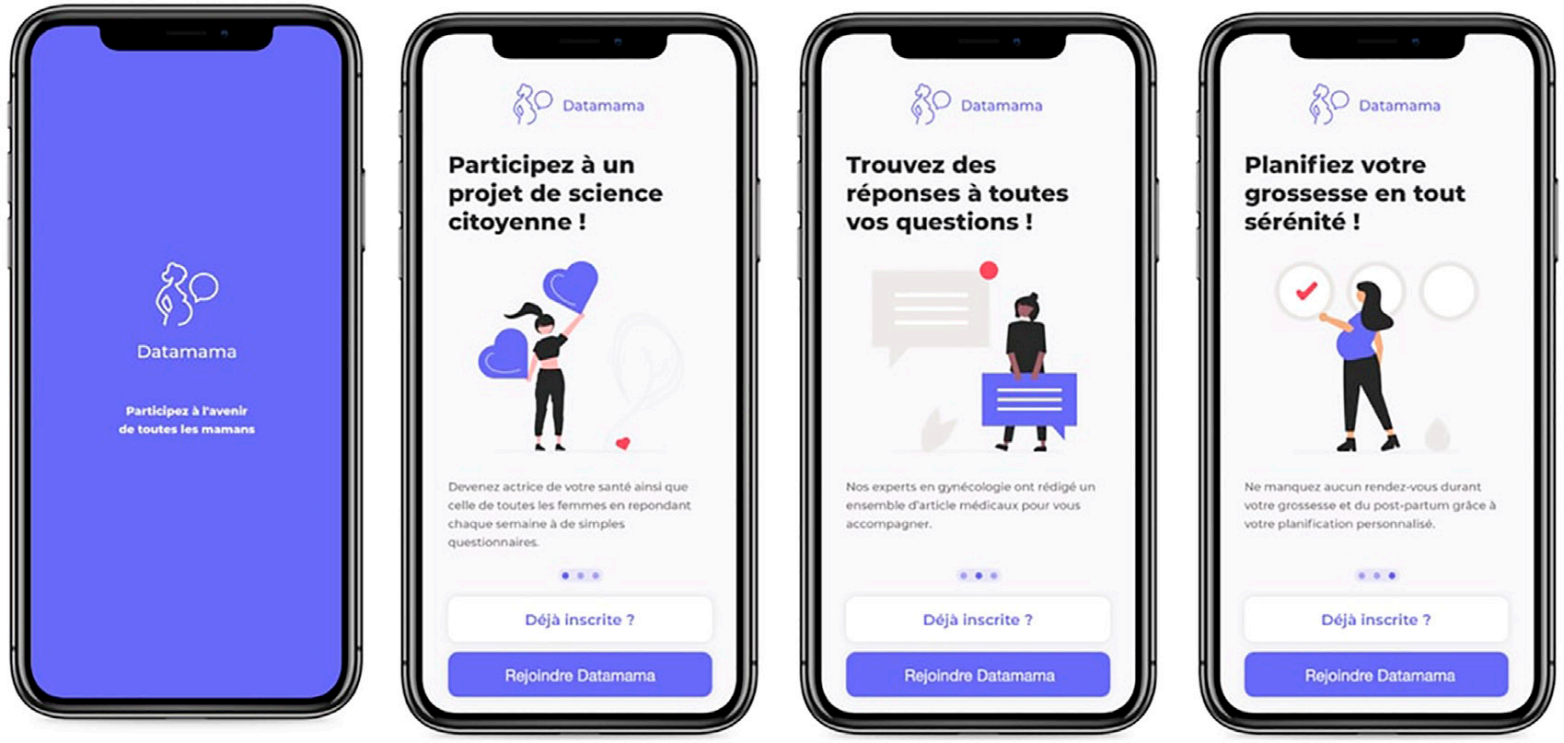
Screenshots of the identity and general design of datamama. The left page shows the loading page; the three next pages are explanations of the app’s purposes before subscription.

The initial name PregRec, a combination of “Pregnancy” and “Recording,” deemed too technical and not sufficiently reflecting the area of pregnancy, and was eventually changed to Datamama. A purple-blue shade was chosen to stand out amid a very pink ecosystem of pregnancy applications. Symbolically, purple has been associated with feminist movements (About International Women’s Day, 2022), and blue with technological innovations and creativity (Marketing and White, 2018). It appeared a perfect illustration of our app’s purpose, i.e., advancing women’s health through technology. A logo was designed to match the new identity. The agency remodeled the initial wireframes to modernise the app and improve user flow at minimal costs. Figure 5 illustrates the identity and general design of Datamama.

### 3.7 Communication and promotion

The efforts of the communication plan have resulted in the acquisition of 122 followers on Instagram, more than 4,000 views on the promotional video following its advertisement by a pregnancy-dedicated page on Instagram, several articles were published in very popular Swiss newspapers [for example, “RTS,” “LFM,” “Tribune de Genève” (rts.ch, 2022; LFM la radio, 2022; de Genève, 2022)] and two radio interviews (RTS and LFM) were conducted. To date, these efforts have resulted in a noticeable increase in subscriptions, with almost 800 users so far.

### 3.8 Usability and feedback

Overall, 65 women completed the first nine questions and 39 of them (60%) provided an answer to the last open-ended question. Most women used the application at least once a week (21/65, 32.3%) to multiple times per week (25/65, 38.5%). One out of five women (14/65, 21.5%) declared having used the application daily, 6.2% (4/65) used it a few times per month and only one woman used it rarely (1/65, 1.5%). Women were then asked to rank the four functionalities of the app according to the extent to which they personally have used it (see Supplementary Material S3). The reason most frequently ranked first was to help research in the field of pregnancy/*postpartum* by answering the questionnaires (42/65, 67% of women rated it first). The second ranked reason to use the app was to obtain the medical information contained in the articles (34/65, 54% of women rated it second), followed by the smart calendar reminding them of pregnancy appointments and exams (35/65, 49% rated it third) and the opportunity to win rewards from the contests (31/65, 56% rated it fourth). Women mostly agreed with the statement that the information given in the medical articles was clear and understandable with a median mark of 9/10 (IQR) = 2) as well as interesting (median mark of 8/10, IQR = 2). Regarding the questionnaires’ frequency, the median mark was 3 (IQR = 3, (0 = “not frequent enough,” 5 = “ideal,” 10 = “too frequent”). Women mostly agreed with the statement that the questionnaires were clear and understandable with a median mark of 8/10 (IQR = 2). Regarding data handling of the information women shared on the app, 98.5% (64/65) of users declared trusting this process. When asked whether they would recommend the app to a friend, the median mark was 8/10 (IQR = 3). Finally, women were asked which functionalities they would like to see developed first in the future. They were given examples of possible answers (see Supplementary Material S2). This open question was completed by 60% of survey users (39/65). Some women may have had multiple suggestions and thus counted as more than one. Responses were grouped into five thematic key concepts (see Supplementary Materials S5, S6 for the French extracts and English translation), being: 1) Medical/psychological information; 2) Questionnaires; 3) New functionalities; 4) Technical issues; and 5) Research communication. The most frequently suggested improvement was related to Medical/psychological information, with women requesting more articles and personalized content based on gestational age, with a weekly/monthly summary of the baby’s development. Suggestions for the questionnaires included receiving more questionnaires and the ability to change answers. New functionalities such as a gestational weight gain tracker, videos and podcasts, and a discussion forum were also proposed. Regarding communication between users and researchers, some women emphasized the importance of sharing the results from the data collected and the desire for direct communication with researchers through the app. Technical issues mentioned included push notifications for new questionnaires and improved app connectivity (extend the time before automatic log out of the app).

## 4 Discussion

This article described the creation, development, promotion, and first feedback of a pregnancy mobile application primarily aimed at collecting data of pregnant women to conduct research. Since its release in June 2022 until today, Feburary 2022, Datamama was downloaded by more than 800 women, demonstrating successful user engagement. However, at the time of writing this paper, we did not have access to the data collected through the app. The reason for this is that the developers have yet to develop the feature that allows for data export and to establish the audit trail that the ethics committee has requested. As a result, further information on the users’ socio-demographics, data completeness and quality cannot be provided yet.

A potential strength and a major differential criterion of our application compared to others available on the market is its reliable content approved by health experts. In a 2019 review of Australian pregnancy apps on Google Playstore which included 76 apps in the final analysis, only 8 (10.5%) declared their affiliations (Brown et al., 2020). In that study, information was the lowest scoring subscale after functionality, esthetics and engagement, based on the Mobile Application Rating Scale (MARS). The authors highlighted the absence of regulation in this field, thus placing the responsibility of including evidence-based information solely on the app developers. Hence, pregnant women may be exposed to inacurrate and even dangerous information (Bland et al., 2020; Brown et al., 2020; Womack et al., 2020). In our survey, women mostly agreed that the information provided on the app was clear and understandable as well as interesting. In fact, the most common suggestion for future improvements of the app in the survey was to increase the content and, more specifically, to add individualised content based on the user’s gestational age, including embryo and fetal development. The need for personalised information based on the stage of pregnancy or the age of the infants was emphasized in 2016 by an Australian focus group on the value of digital media for pregnancy and early motherhood information (Lupton, 2016). Interactive apps in which the information was personal to the woman’s own pregnancy were the most popular category of apps based on a review of pregnancy apps in the Appstore and Playstore in 2014 (Tripp et al., 2014). According to the authors of this review, this type of functionality allows to mimic the interaction of a healthcare consultation. Thus, efforts towards regularly updating and adding new content should be made to keep pregnant women interested. Priority will be given to address the topic of fetal development and expand the content related to infant health beyond the first 8 weeks of *postpartum*, as suggested by some of the survey’s users.

As part of the development phase, specific measures have been taken to encourage data collection and accuracy in the app such as the architecture of the questionnaires (i.e., via a chatbot function) and the gamification strategy. Results from the survey showed that the majority of women were satisfied with the frequency of the questionnaires and perceived them as clear and understandable. A few women asked to be able to change their answers to the questionnaires when they made a mistake, which shall be considered in the future version of the app. Push notifications to inform users of new questionnaires or available medical articles is another feature that will soon be implemented to keep users engaged. Not only were push notifications requested by women in the survey, they have also been recognized as a valuable feature of pregnancy apps providing timely information to users and enhancing the sense of interactivity (Lupton, 2016; Halili et al., 2018). This has been observed both in the Australian focus group (Lupton, 2016), as well as among users of the Canadian SmartMoms app (Halili et al., 2018).

One major challenge in the development consisted of ensuring that data are securely collected, stored, and manipulated. This is not only a legal requirement but is also a real preoccupation for mobile apps users who have highlighted lack of trust in data storage and privacy as potential barriers to app usage (Bert et al., 2016; Burgess et al., 2017; Biviji et al., 2021). All survey respondents, with the exception of one, trusted the app to handle the data they shared. It is likely that the endorsement and support of the Lausanne University Hospital contributed to the trust placed in Datamama since most pregnancy apps are developed by internet portals and commercial companies (Lee and Moon, 2016). However, another explanation would be that only women who trusted the app enrolled in it, resulting in selection bias among app users.

To ensure this retention of users, future developments have been planned to align with the suggestions made by women in the survey. First, these developments include new features, of which some were initially proposed but could not be immediately integrated due to limitations in time and budget. One such feature is a gestational weight gain tracker, also suggested in the survey, which has the potential to improve women’s gestational weight control and lower the risk of obstetrical complications (Sandborg et al., 2021). In addition, it would likely increase the accuracy of the data we collect on this parameter. Other health parameters could be more accurately measured by linking Datamama to connected devices and other health monitoring applications (i.e., related to nutrition, physical activity or sleep monitoring). A discussion forum was surprisingly only mentioned once in the survey although it is a very popular functionality of pregnancy apps (Goetz et al., 2017; Sommer et al., 2017; Halili et al., 2018). Indeed, women typically report feeling supported by sharing their experience with other expectant mothers. A forum was initially considered, but ultimately not implemented due to monitoring considerations.

Finally, one potential limitation of Datamama relates to the risk of selection bias. Indeed, participants may have been skewed towards healthy, young pregnant women of higher socio-economic status who actively engaged with the app, participated in gaming challenges, and filled in questionnaires. This group may not be representative of all pregnant women, particularly those who are socially disadvantaged, suffer from depression, eating disorders, or drug addiction, and may not have the same level of access to resources or interest in participating in the study. In the future, to determine whether our data reflect a representative sample of Swiss pregnant women, the users’ socio-demographic characteristics will be compared to those of Swiss pregnant women. Furthermore, a “drug utilization” study on medication use during pregnancy within our cohort will be conducted to compare these results with previous studies on the subject (Gerbier et al., 2021; Gerbier et al., 2022).

## 5 Conclusion

Datamama is the first citizen-science based pregnancy application serving both patients and researchers needs. Initial feedback from the users was highly positive and highlighted the future challenges that should be addressed to ensure its success. Future work will consist of using the data collected to test its validity and answer specific pregnancy-related questions.

## Data Availability

The original contributions presented in the study are included in the article/Supplementary Materials, further inquiries can be directed to the corresponding author.

## Supplementary material

SUPPLEMENTARY MATERIAL S1 PRISMA flow diagram describing the literature search through Pubmed and the mobile application search through the Swiss AppStore search.
