# Supplementary material for: Datamama, bringing pregnancy research into the future: design, development, and evaluation of a citizen science pregnancy mobile application
Source: Front Drug Saf Regul. 2023 May 4;3:1187023. doi: 10.3389/fdsfr.2023.1187023 (PMC12443117; doi:10.3389/fdsfr.2023.1187023)
Supplement: Supplementary file 1 [file Table1.docx]

**Supplementary Materials**

**Supplementary Material S2:** French version of the survey on women's use and expectations regarding the pregnancy mobile application Datamama.

**Supplementary Material S3:** English translation of the survey on women's use and expectations regarding the pregnancy mobile application Datamama.

**Supplementary Material S4:** List of the 25 apps reviewed on the AppStore.

**Supplementary Material S5:** French extracts from the responses to the open question regarding the future developments of the app grouped into five thematic key concepts.

**Supplementary Material S6:** English translation of the extracts from the responses to the open question regarding the future developments of the app grouped into five thematic key concepts.

**Supplementary Material S2:** French version of the survey on women's use and expectations regarding the pregnancy mobile application Datamama.

| **Questions** | **Answer type and choice** |
| --- | --- |
| À quelle fréquence utilisez-vous l'application ? | Multiple choice:  1. Tous les jours ;  2. Plusieurs fois par semaine ;  3. Environ une fois par semaine;  4. Quelques fois par mois ; 5. Rarement |
| Pour quelles raisons utilisez-vous principalement Datamama ? | Ranking question:  1. Les questionnaires pour faire avancer la recherche dans le domaine de la grossesse / post-partum.  2. L'information médicale sous forme d'articles  3. Le calendrier me rappelant les différents rendez-vous et examens que je dois réaliser.  4. La possibilité de remporter des récompenses grâce aux concours. |
| Veuillez noter à quel point vous êtes d'accord avec cette affirmation (0 pas du tout d'accord, 10 tout à fait d'accord): "L'information des articles médicaux est rédigée de manière claire et compréhensible." | Rating scale from 0 to 10 |
| Veuillez noter à quel point vous êtes d'accord avec cette affirmation (0 pas du tout d'accord, 10 tout à fait d'accord): "Le sujet des articles médicaux vous intéresse." | Rating scale from 0 to 10 |
| Que pensez-vous de la fréquence des questionnaires ?  Attention, dans cette question, 5 correspond à une fréquence idéale. | Rating scale from 0 to 10 (0 "pas assez fréquent", 5 "idéale", 10 "trop fréquent") |
| Veuillez noter à quel point vous êtes d'accord avec cette affirmation (0 pas du tout d'accord, 10 tout à fait d'accord): "Les questionnaires sont clairs et compréhensibles." | Rating scale from 0 to 10 (0 "pas clair du tout", 10 "tout à fait clair") |
| Avez-vous confiance dans le traitement des données que vous partagez sur Datamama ? | Yes/no type |
| Veuillez noter à quel point vous êtes d'accord avec cette affirmation (0 pas du tout d'accord, 10 tout à fait d'accord): " Recommanderiez-vous l'application à une amie ? | Rating scale from 0 to 10 (0 "pas du tout", 10 "Fortement") |
| À l'avenir, quelles fonctionnalités aimeriez-vous voir développer en priorité sur Datamama ?  (Par exemple, augmentation des articles médicaux, suggestions de thèmes d'articles, nouvelles fonctionnalités, etc) | Open ended question |

**Supplementary Material S3:** English translation of the survey on women's use and expectations regarding the pregnancy mobile application Datamama.

| **Questions** | **Answer type and choice** |
| --- | --- |
| How often do you use the application? | Multiple choice:  1. Everyday;  2. Multiple times per week;  3. Approximately once a week;  4. A few times per month;  5. Rarely |
| For which reason do you mainly use the application? | Ranking question:  1. The questionnaires to advance research in the field of pregnancy/post-partum.  2. The medical information availble in the articles.  3. The calendar reminding me of the different appointments and exams I have to attend.  4. The possibility to win a reward with the contests. |
| Please rate to which extent you agree with the following sentence (0 not at all, 10 completely agree): "The medical information is written in a clear and understandable manner." | Rating scale from 0 to 10 |
| Please rate to which extent you agree with the following sentence (0 not at all, 10 completely agree): "The topics of the medical articles are interesting." | Rating scale from 0 to 10 |
| What do you think of the questionnaires frequency ?  Beware that in this question, 5 corresponds to an ideal frequency. | Rating scale from 0 to 10 (0 "not frequent enough", 5 "ideal", 10 "too frequent") |
| Please rate to which extent you agree with the following sentence (0 not at all, 10 completely agree): "The questionnaires are written in a clear and understandable manner." | Rating scale from 0 to 10 (0 "not clear at all", 10 "perfectly clear") |
| Do you trust the handling of the data you share on Datamama? | Yes/no type |
| Please rate to which extent you agree with the following sentence (0 not at all, 10 completely agree): " Would you recommend the app to a friend ? | Rating scale from 0 to 10 (0 "not at all", 10 "Strongly") |
| In the future, which functionalities would you like to see developed first on Datamama?  (For example, increase the number of articles, new topics suggestions, new functionalities, etc) | Open ended question |

**Supplementary Material S4:** List of the 25 apps reviewed on the AppStore.

1) 25 most popular apps on the AppStore: "Grossesse +"; "WeMoms"; "Alimentation grossesse"; "Baby center"; "Grossesse et suivi de enceinte"; "Contractions de grossesse"; "Materniteam"; "Grossesse by Parents"; "ma grossesse doctissimo"; "Beebs"; "iMaman"; "moi bientôt maman"; "neomama"; "YooMum"; "Enceinte: ma grossesse et moi"; "Enceinte: que puis-je manger?"; "Mon alimentation grossesse"; "calendrier de grossesse"; "Ma grossesse"; "neuf mois"; "la boîte rose"; "Enceinte quoi manger"; "âge gestationnel"; "Cocoon life".

**Supplementary Material S5:** French extracts of the responses to the open question regarding the future developments of the app grouped into five thematic key concepts.

| **Questionnaires** | **Medical information** | **New functionalities** | **Research communication** | **Technical issues** |
| --- | --- | --- | --- | --- |
| Possibilité de changer notre réponse en cas d'erreur | Plus d'information sur le post-partum | Document permettant de suivre sa courbe de poids | Possibilité de poser des questions | Notifications lors de nouveaux questionnaires |
| Les questionnaires posent toujours les mêmes questions | Plus d'articles médicaux | Chat entre utilisateurs | Lien vers des articles scientifiques, statistiques, retour sur l'utilisation des données | Rester connectée |
| Plus de questionnaires | Info sur bébé par semaines ou mois, info jumeaux | Vidéos, podcasts | Pouvoir mettre que l'on a fait une fausse couche | Résolution des problèmes de connexion à l'app |
|  | Plus d'articles liés au moment de la grossesse en cours. Ex: "femme à 8 semaines d'aménorrhée= un article sur la nourriture autorisée*, plus cibler le moment. |  |  |  |

**Supplementary Material S6:** English translation of the responses to the open question regarding the future developments of the app grouped into five thematic key concepts.

| **Questionnaires** | **Medical information** | **New functionalities** | **Research communication** | **Technical issues** |
| --- | --- | --- | --- | --- |
| Possibility to change an answer | More information on post-partum | Gestational weight gain tracker | Possibility to ask questions | Notifications when new questionnaires are available |
| They are redundant and ask the same questions | More medical articles | Discussion forum | Feedback on how the data are used | Stay logged in |
| More questionnaires | Weekly information on the baby and information on twins | Podcasts and videos | Being able to tell that a person has miscarried | Connection issues |
|  | Information should be tailored to the gestational age |  |  |  |
